# Large vessel vasculopathy: An underrecognized complication in Wiskott–Aldrich syndrome

**DOI:** 10.70962/jhi.20250242

**Published:** 2026-05-26

**Authors:** Deepti Suri, Pallavi L. Nadig, Dev Desai, Yamini Sharma, Ahmed Jamal, Gayathri C. Vaitheeswaran, Himanshi Choudhary, Murugan Sudhakar, Ridhima Aggarwal, Suprit Basu, Pratibha Suku, Vibhu Joshi, Rashmi Rikhi, Anmol Bhatia, Akshay Saxena, Manphool Singhal, Manpreet Dhaliwal, Saniya Sharma, Rakesh Kumar Pilania, Ankur Kumar Jindal, Vignesh Pandiarajan, Amit Rawat, Anju Gupta, Surjit Singh

**Affiliations:** 1 Pediatric Allergy Immunology Unit, Department of Pediatrics, Postgraduate Institute of Medical Education & Research, Chandigarh, India; 2 Department of Radiodiagnosis and Imaging, Postgraduate Institute of Medical Education & Research, Chandigarh, India

## Abstract

Autoimmune manifestations occur in 25–75% of patients with Wiskott–Aldrich syndrome (WAS), commonly including autoimmune hemolytic anemia, skin vasculitis, IgA nephropathy, arthritis, and inflammatory bowel disease. Large vessel vasculopathy is rarely reported in WAS. We present a review of children with WAS and evidence of large vessel vasculopathy at our center, analyzing clinical, radiological, immunological, and genetic data. Among 80 patients diagnosed with WAS over two decades, four children (aged 10–18 years) developed large vessel vasculopathy. Two had classical WAS with bleeding, recurrent infections, and eczema since infancy, while two had milder phenotypes and presented for the first time during this illness. Clinical features included chest pain and heart failure, abdominal pain, upper limb claudication, and differential blood pressure. Imaging demonstrated aneurysmal dilatation of the aorta and its major branches. Epstein-Barr virus viremia was detected in three patients. All received intravenous immunoglobulin and immunosuppressive therapy; none could undergo hematopoietic stem cell transplantation (HSCT), and three patients died. Large vessel vasculopathy is a rare but life-threatening complication of WAS, underscoring the importance of early recognition and timely consideration of HSCT.

## Teaser

Suri et al. from PGIMER, Chandigarh, India, report large vessel vasculopathy as a rare, life-threatening, and underrecognized complication of Wiskott–Aldrich syndrome, often mimicking Takayasu arteritis. They implicate immune dysregulation, endothelial injury, and viral triggers, emphasizing early vascular screening and timely definitive therapy to reduce high mortality.

## Introduction

Patients with Wiskott–Aldrich syndrome (WAS) are at an increased risk for developing autoimmunity and malignancy. Although the exact mechanism for this increased susceptibility remains incompletely understood, around 30–70% of patients develop some form of autoimmune disease, especially if they do not undergo hematopoietic stem cell transplantation (HSCT) (1). Reported autoimmune complications include autoimmune cytopenia, vasculitis, arthritis, inflammatory bowel disease, and IgA nephropathy. Among these, vasculitis is the second most common manifestation. While small vessel vasculitides such as Henoch–Schoenlein purpura and IgA nephropathy are more frequently reported (2, 3), large vessel vasculitis remains an extremely rare, but life-threatening, complication.

This case series aims to describe the clinical, radiological, and genetic features of four pediatric patients diagnosed with WAS who developed large vessel vasculopathy. It is highlighted that in two of these four patients, initial diagnosis of Takayasu arteritis was established; however, persistent thrombocytopenia prompted screening for WAS.

## Results

Of the 80 patients with WAS registered at our center, in our follow-up clinic, four patients were identified to have large vessel vasculopathy resembling Takayasu arteritis. Details of these patients are as follows.

### Patient 1 (2020)

**Figure 1.**
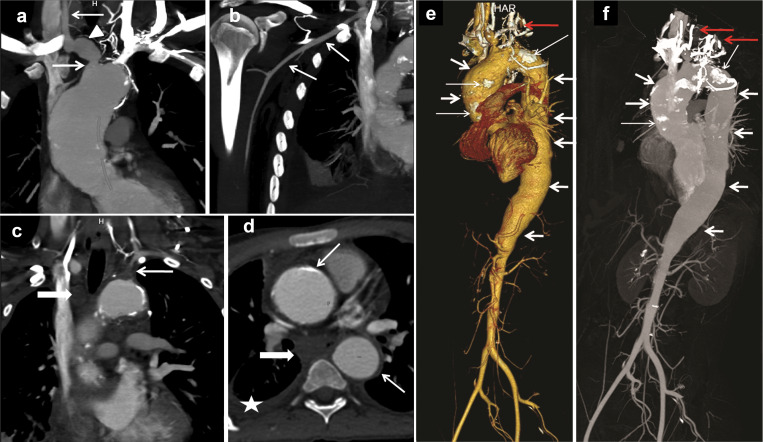
**Panel of CT angiography images showing aneurysmal dilataion of aorta and its branches with development of collaterals and mediatstinal lymphadenopathy in patient 1. (a–d)** Panel of CT angiogram images of patient 1: reconstructed coronal images (a and b) show focal stenosis at the origin of the right brachiocephalic trunk (solid white arrow below the arrowhead) with poststenotic dilation (arrowhead). Right common carotid (solid white arrow above the arrowhead in a) and subclavian (arrows in b) are normal. Reconstructed coronal image (c) shows nonopacified (occluded) proximal left subclavian artery until the origin of the vertebral artery (arrow). Note the left common carotid artery is also not opacified. Conglomerate necrotic lymph nodes are seen in right paratracheal location (thick arrow in c). Axial image (d) shows circumferential mural thickening of the ascending and descending thoracic aorta with mural calcifications (thin arrows). Note necrotic subcarinal lymphadenopathy (thick arrow) and right pleural effusion (asterisk). **(e and f) **CT angiogram volume rendered (e) and maximum-intensity projection (f) images show aneurysmal dilatation of the aorta extending from the aortic root up to the suprarenal part of the abdominal aorta (thick white arrows). Note multiple mural calcifications (thin white arrows) and multiple collaterals in the neck (red arrows). Visceral arteries including renal and iliac arteries are normal.

A 5-year-old boy presented with complaints of acute febrile encephalopathy, seizures, and acute-onset deviation of angle of the mouth to the left. He had a history of recurrent sinopulmonary infections, generalized eczematous rash, recurrent epistaxis, and skin bleeds since infancy. Examination revealed petechiae, eczema, generalized lymphadenopathy, hepatosplenomegaly, and signs of meningeal irritation with bilateral lateral rectus palsy and right-sided lower motor neuron facial nerve palsy. Cerebrospinal fluid analysis was cellular with hypoglycorrhachia, but culture was sterile (Table 1). Magnetic resonance imaging (MRI) brain did not reveal any intracranial bleed. In view of history of recurrent infections with persistent thrombocytopenia, the possibility of WAS was considered. The WAS protein (WASP) expression was reduced, and genetic testing revealed a pathogenic variant in the *WAS* gene (c.1021+2T>G [intron 10, IVS10+2]). He improved following treatment with intravenous ceftriaxone. Replacement intravenous immunoglobulin (IVIg) therapy and evaluation for hematopoietic stem cell transplantation (HSCT) were advised. However, the child was lost to follow-up. He continued to have intermittent bleeding episodes, recurrent otitis media, and pulmonary infections, which were managed at a local health care facility with antimicrobials. He was readmitted to our institute at 16 years of age with complaints of intermittent, severe left chest pain and congestive heart failure. Examination revealed absent pulsations in the left upper limb arteries with differential blood pressures (left upper arm supine: not recordable due to lower limb hypertension), peripheral signs of aortic regurgitation, and hepatosplenomegaly. Investigations are summarized in Table 1. Computed tomography (CT) angiogram showed complete occlusion of left subclavian artery and left common carotid (with collaterals), aneurysmal dilatation of aorta extending from the aortic root up to suprarenal part of the abdominal aorta, and right main renal artery narrowing at the ostium (Fig. 1). He was also noted to have left lower lobe consolidation, right-sided pleural effusion, and necrotic mediastinal lymphadenopathy. Positron emission tomography was suggestive of increased uptake in the cervical, supraclavicular, mediastinal, axillary, and abdominopelvic lymph nodes, omental–serosal deposits, and pleural effusion. Epstein-Barr virus (EBV) viral load was high in the blood; a possibility of EBV-associated lymphoproliferation or lymphoma or disseminated tuberculosis was kept. The clinical condition rapidly deteriorated and developed altered sensorium and acute right hemiparesis within a week of admission. MRI brain showed evidence of multiple microhemorrhages suggesting vasculitic infarcts. He was initiated on corticosteroids with rituximab (375 mg/m^2^) as rescue therapy but had progressive neurological deterioration and eventually succumbed to the illness.

**Table 1.** Summary of clinical characteristics of four patients with WAS gene mutation with large vessel vasculopathy in our cohort

​	Patient 1	Patient 2	Patient 3	Patient 4
Age of presentation	5 years	3.5 years	11 years	11.8 years
Age of onset of initial symptoms	Infancy	2 years	8 years	11.4 years
Age at diagnosis of WAS	5 years	3.5 years	11 years	11.8 years
Age at the time of vasculitis manifestations	16 years	10 years	11 years	11.4 years
Presenting complaints	Acute febrile encephalopathy, seizures, right-sided lower motor neuron facial nerve palsy, bilateral lateral rectus palsy	Recurrent abdominal pain	Polyarthritis, recurrent abdominal pain, cough, hypertension, cardiac dysfunction	Abdominal pain, neck pain, left upper limb claudication
Lymphoproliferation	Present	Present	Present	Absent
**Past infections**				
Bacterial infections	Sinopulmonary infections, pyogenic meningitis, pulmonary Tuberculosis	Sinopulmonary infections	None	None
Viral infections	None	Warts, extensive molluscum contagiosum	None	None
Autoimmune manifestations, if any	DCT negative autoimmune hemolytic anemia	Autoimmune hemolytic anemia	Arthritis, ANA negative, DCT negative	None
Eczema	++	++	−	−
IVIg prophylaxis at the time of vasculitis	Noncompliant	Noncompliant	Not initiated	Not initiated
EBV viral load	9720 copies/ml	38,880 copies/ml	Negative	5200 copies
CMV PCR, Serology:(HIV, HSV, HCV) and HBsAg	Negative	Negative	Negative	Negative
**Investigations**				
MPV (fl)	6.7	6.8	7.4	6.1
Lowest platelet counts at presentation with vasculitis (×10^9^/L)	2.9	35	69	136
**Immunoglobulin profile**				
IgG (g/L) (N:5–16)	12.4	7.5	**6.67**	14.9
IgM (g/L) (N:0.5–2)	1.35	**0.24**	**0.47**	0.50
IgA (g/L) (N:0.4–2)	0.59	**5.68**	1.92	**5.83**
IgE (KU/L; N<100)	**370**	**6720**	**786**	**1256**
**Lymphocyte Subset analysis**				
CD3^+^ T lymphocytes (N: 55–75%)	61.9%	76.64%	**56.9%**	**86.5%**
CD19^+^ B lymphocytes (N: 10–30%)	**7.6%**	**4.99%**	**30.58%**	**5.09%**
CD16/56^+^ NK cells (N:4–23%)	7.3%	10.69%	11.81%	7.14%
CD3^+^ CD4^+^ T cell (N:27–53%)	36.25%	36.25%	**69.75%**	**71.6%**
CD3^+^ CD8^+^ T cell (N: 19–34%)	**55.97%**	**55.97%**	21.14%	**19.4%**
CD4:CD8 ratio (N: 0.9–3.6)	**0.65**	**0.65**	3.3	**4.1**
CD4^+^ CD127^–^ CD25^+^ (T_regs_) (N: 5–10%)	4%	4%	**14.45%**	8.8%
**WAS protein expression**				
Delta MFI	Patient: 1200	Patient: 281	Patient: 1495	Patient: 5128
	Control: 4300	Control: 1087	Control: 4761	Control: 7875
Stain index	Patient: 4	Patient: 4.17	Patient: 159	Patient: 145.46
	Control: 15.33	Control 8.68	Control: 428	Control: 65.68
WAS protein	Reduced	Reduced	Reduced	Normal
*WAS* gene variant	c.1021+2T>G, Intron 10-IVS 10+2 T>G	Exon 1 c.37C>T; p.R13*	Exon 2 c.256C>T, p.Arg86Cys	Exon 1 c.103 C>T, p.Leu35Phe
Arterial involvement	Thoracic and suprarenal part of abdominal aorta, critical focal luminal narrowing of brachiocephalic trunk at its origin with complete luminal occlusion of left subclavian artery and left common carotid artery	Abdominal aorta extending from diaphragmatic hiatus to bifurcation (L5 vertebral level). Variable length of proximal coeliac trunk, superior mesenteric artery and left renal artery showed fusiform dilatations	Suprarenal and renal segments and short infrarenal segment of the abdominal aorta, celiac artery origin, anterior right renal artery, posterior right renal artery	Long segment occlusion / tight stenosis of left subclavian artery.

Bold values indicate abnormal values.

N, age related normal values; WAS, Wiskott Aldrich syndrome; DCT, direct coombs test; fl, femtoliter; ANA, Anti nuclear antibody; IVIg, intravenous immunoglobulin; NK cell, natural killer cells, T_regs_, Regulatory T cells; MFI, mean fluorescence index; EBV, Epstein barr virus; PCR, polymerase chain reaction, HIV-Human immunodeficiency virus; HSV, herpes simplex virus; HCV, Hepatitis C Virus; HBsAg, Hepatitis B surface antigen.

### Patient 2 (2022)

**Figure 2.**
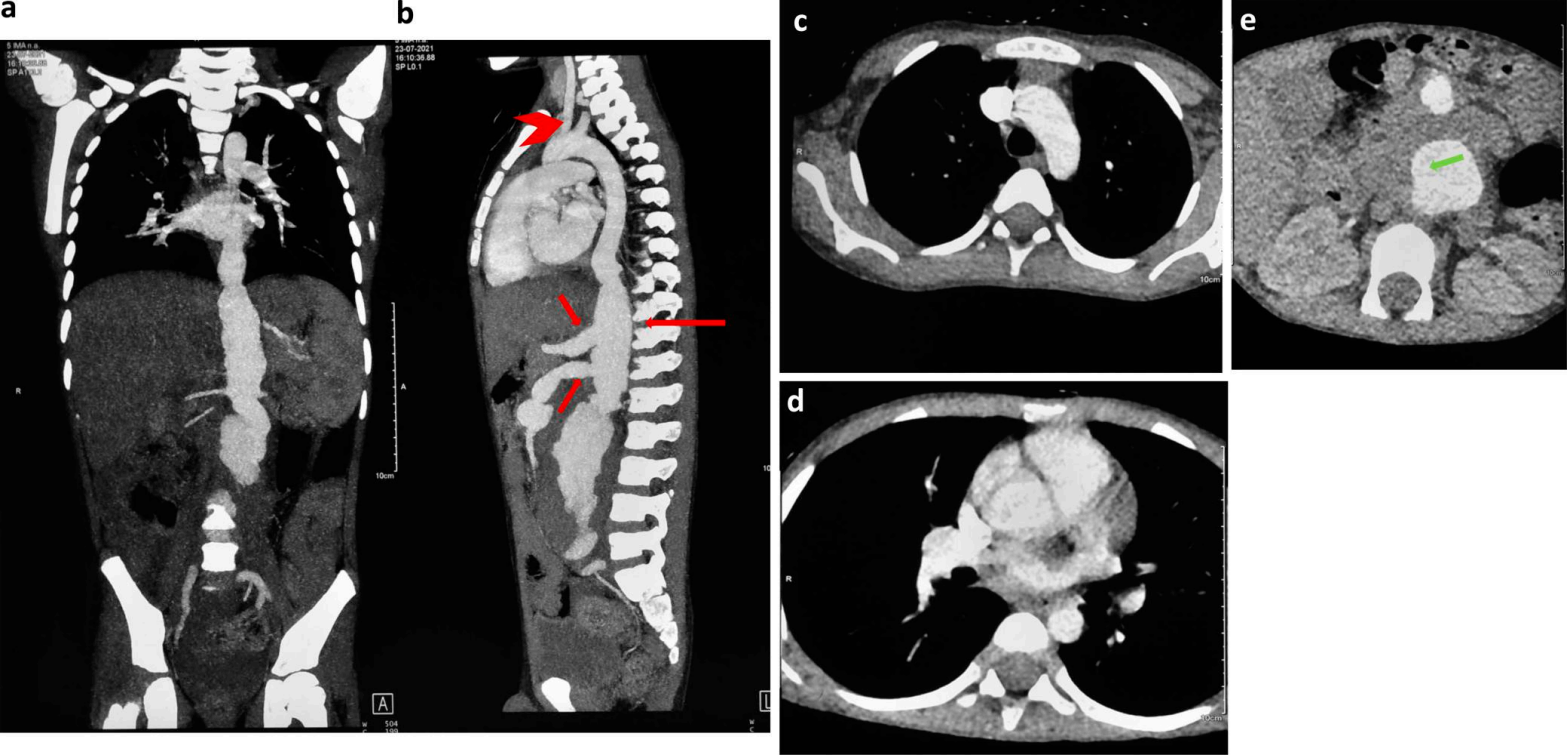
**Panel of CT angiography images demonstrating vascular involvement of thoracic and abdominal vessels in Patient 2. (a–e)** CT angiography of the thorax and abdomen of patient 2: coronal (a), sagittal (b), and axial (c–e) sections demonstrating the fusiform and irregular outline of the abdominal aorta (long red arrow in b) extending from the diaphragmatic hiatus to its bifurcation at the L5 vertebral level with a maximum transverse caliber of 2.7 cm. A thick rim of hypodense soft tissue is seen surrounding the aneurysmal segment suggestive of partial thrombosis (green arrow mark). Variable lengths of proximal parts of the celiac trunk (upper short red arrow in b), superior mesenteric artery (lower short red arrow in b), and left renal artery show fusiform dilatation. Aortic root, ascending aorta, aortic arch (c) and its branches (arrowhead), and descending thoracic aorta are normal in course, caliber, and contrast opacification. The two renal arteries are seen on the right side, and the inferior mesenteric artery is normal in course, caliber, and outline.

A 3.5-year-old boy was diagnosed to have WAS when he presented with blood-stained diarrhea since the neonatal period. There was family history of multiple male infant deaths. He was negotiated for HSCT; however, it could not be proceeded further due to the nonavailability of a matched donor and financial constraints. He continued to have intermittent bleeding episodes and infections despite replacement IVIg and cotrimoxazole prophylaxis. At the age of 10 years, he presented with complaints of abdominal pain for 7 days and vomiting. Examination revealed cervical lymphadenopathy, ear discharge with perforation of the tympanic membrane, eczematous lesion over the body with extensive molluscum contagiosum, differential pulses and differential blood pressures between upper limbs and lower limbs, and pulsatile mass in the epigastric region. Laboratory investigations (summarized in Table 2) revealed elevated inflammatory markers. Ultrasonographic assessment of the abdomen revealed a fusiform dilatation in the abdominal aorta, which was later confirmed by CT angiography with a maximum transverse caliber of 27 mm, extending from the diaphragmatic hiatus to its bifurcation. Variable lengths of proximal parts of the coeliac trunk, smooth muscle actin (SMA), and left renal artery show fusiform dilatation (Fig. 2). Pus from the ear showed growth of *Pseudomonas aeruginosa*, which was treated with sensitive antibiotics for 2 wk. Viral serologies (herpes simplex virus [HSV], hepatitis B virus [HBV], hepatitis C virus [HCV] IgM) and workup for tuberculosis were negative. EBV viral load was found to be elevated (38,880 copies/ml). He received pulse methylprednisolone and IVIg and was initiated on oral methotrexate, warfarin for large vessel vasculopathy, and was reinitiated on prophylactic IVIg and cotrimoxazole. On follow-up at 4 mo, he developed acute abdominal pain with diarrhea and was detected to have a thrombus in the aneurysmal segment of the abdominal aorta. Methylprednisolone pulses reinitiated along with anticoagulation with low molecular weight heparin, however, succumbed to illness.

**Table 2.** Review of literature of previously described cases of WAS with large vessel vasculopathy

Study (year)	No. of patients	Age at diagnosis	Age at developing vasculitis	Symptoms and signs	Type of vasculitis	Infections	Treatment	Outcome	Relapse
Ilowite et al. (1986) (4)	1	9 mo	12 years	Fever, chest pain, dyspnea, differential pulses	Lymphomatoid granulomatosis pulmonary vasculitis, calcified aortic ring, mitral and aortic regurgitation, partially obstructed left subclavian artery	EBV	Methylprednisolone, gentamycin, acyclovir, oral cyclophosphamide	Death after 2 years following pneumonia	​
Lau et al. (1992) (5)	1	4.5 years	5.5 years	Abdominal pain, hypertension, hypertensive encephalopathy, differential renal sizes	Abdominal aortic aneurysm, stenosis of bilateral renal arteries	Nil	Renal autotransplant	Death at 5.5 years due to ventricular arrhythmia	​
Son et al. (1995) (6)	1	–	23 years	Incidentally detected mediastinal widening in a chest roentgenogram	Aortic insufficiency, ascending and descending aorta, t horacic dilatation	Nil	Surgical repair	Recovered	Relapse at 9-year follow-up
Cluggage et al. (1999) (7)	1	–	24 years	Abdominal pain, melena, hematemesis, hypertension, gastrointestinal hemorrhage	Multiple aneurysms from distal branches of right and left hepatic arteries, superior mesenteric artery, perirenal hematoma	Nil	Nephrectomy	Recovered, well after 7 wk	​
Johnston et al. (2001) (8)	1	6 years	17 years	Signs of aortic regurgitation	Dilatation of the ascending aorta, aortic arch, and proximal descending aorta	Nil	Surgical repair	Recovered, well after 2 years of follow-up	​
Narayan et al. (2004) (9)	1	6 years	24 years	Shortness of breath	Severe aortic regurgitation, dilatation of the aortic root and ascending aorta	Nil	Surgical repair	Recovered, well in 1-year follow-up	At 3 years, requiring second surgery
Bernabeau et al. (2007) (10)	1	15 years	33 years	Incidentally detected mediastinal widening in a chest roentgenogram	Thoracic aortic aneurysm involving ascending aorta, arch, and descending thoracic aorta	Nil	Surgical repair	Recovered, well in 10-mo follow-up	​
Faganello 2008 (11)	1	NA	27 years	–	Aortic root, ascending aorta	NA	Two-staged surgical repair	Developed aortobronchial fistula and massive hemoptysis and succumbed	​
Ono et al. (2009) (12)	1	5 years	7 years	Incidentally detected in a routine chest roentgenogram	Ascending aortic aneurysm and moderate aortic regurgitation	Nil	Surgical repair	Recovered, well at 5-mo follow-up	​
Pellier et al. (2010) (13)	5	​	Mean age: 12.6 years	4 asymptomatic 1 acute chest pain	Case 1: arch and descending thoracic aorta Case 2: descending thoracic aorta Case 3: abdominal aorta Case 4: descending thoracic aorta and another 3 cm lower of aortic wall Case 5: ascending thoracic aorta	Case 5: VZV, EBV, and HHV-6 in the aortic material	Case 1: HSCT Case 2: HSCT Case 3: nil Case 4: nil Case 5: surgical repair, steroids and methotrexate for 18 mo	Case 1: progression of aneurysm, asymptomatic after 5 years Case 2: death due to herpes simplex viral encephalitis after 4 years of diagnosis Case 3: died sue to lymphoma after 2 years Case 4: asymptomatic, aneurysm size stable over 12 years of follow-up Case 5: dysplastic aorta without clinical symptoms at 6 years of follow-up	​
Önalan et al. (2018) and Önalan et al. (2023) (14, 15)	1	10 years	12 years	Chest pain and dyspnea	Descending aorta at 12 years of age At 21 year**s **(9 years following HSCT), ascending aortic aneurysm with aortic valve insufficiency	Nil presence of an aortitis with granulomatous inflammatory response and multinucleated cells	Surgical repair	Ascending aortic aneurysm at 21 years, surgically operated Alive at 21 years of age	Nil

EBV, Epstein-Barr virus; VZV, varicella-zoster virus; HHV-6, human herpesvirus 6; HSCT, hematopoietic stem cell transplantation.

### Patient 3 (2023)

**Figure 3.**
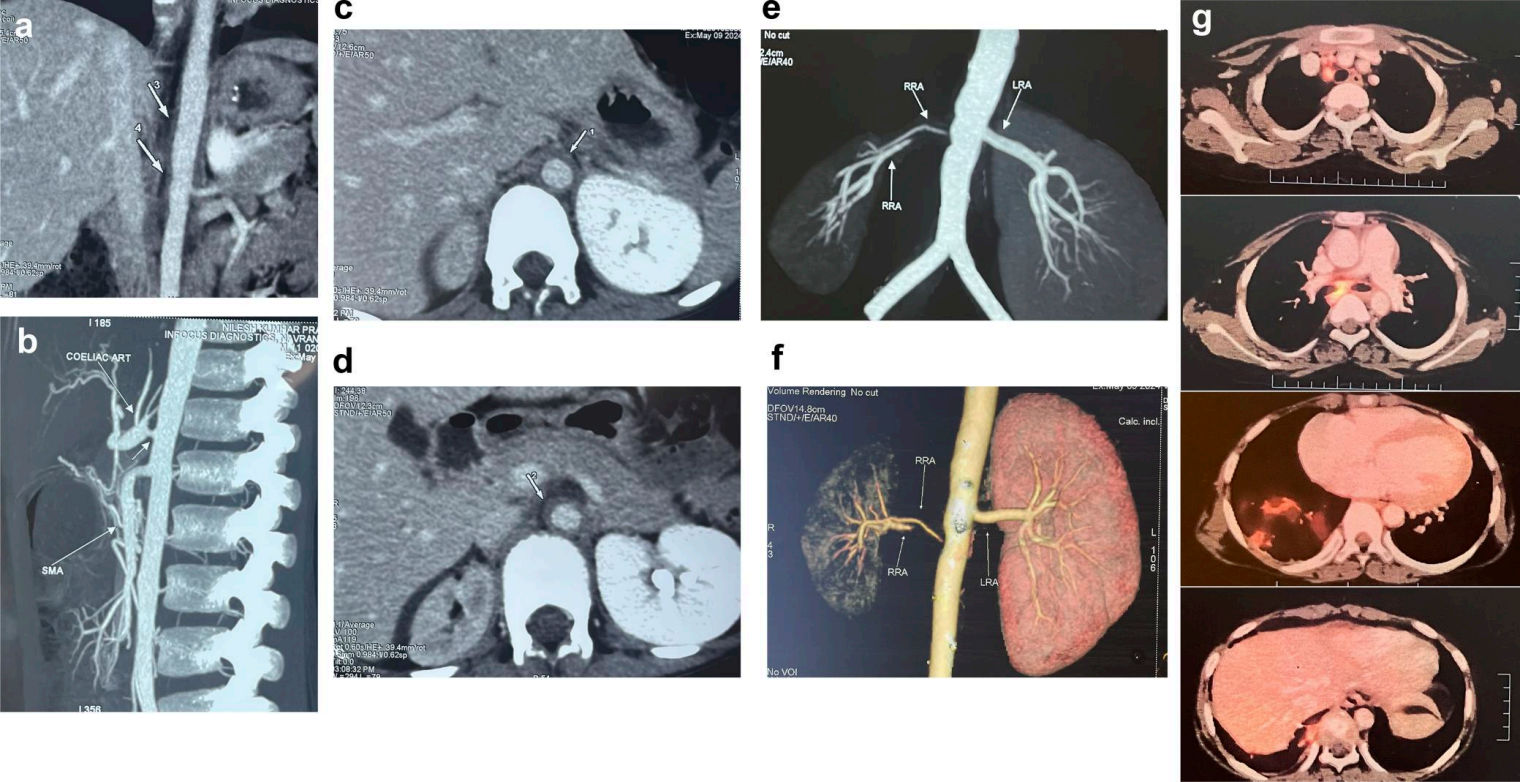
**Panel of CT angiogram images showing vascular involvement and stenotic lesions and PET scan revealing non-necrotic lympadenopathy in patient 3. (a, c, and d)** CT angiography of abdominal arteries of patient 3 shows concentric wall thickening from the level of the aortic hiatus, the entire suprarenal and renal segments (arrows labelled as 1 to 4 in a to d), and the short infrarenal segment of the abdominal aorta for a length of 9.0–9.5 cm and maximum wall thickness of 3.2 mm. **(b–d)** Mild eccentric narrowing at the celiac artery origin of about 20–30% stenosis, severe short-segmental stenosis/near-total occlusion at the origin of the anterior right renal artery (c), and long segmental occlusion for a length of 14 mm of the posterior right renal artery (d). The rest of the infrarenal abdominal aorta, aortic bifurcation, and both iliac arteries appear normal in course, caliber, outline, and branching pattern. **(e and f)** Right kidney (measures 7.5 cm) shows mild diffuse shrinkage in volume with mild hypoperfusion of the right kidney with delayed excretion, whereas the left kidney (measures 11.1 cm) shows compensatory enlargement, and normal perfusion and normal excretion. **(g)** Positron emission tomography scan shows no evidence of abnormal FDG uptake at concentric wall thickening/stenosis involving abdominal aorta and its branches. Multiple FDG avid patchy consolidation changes are seen mainly in the subpleural and few in the peribronchial regions of the lower aspect of the right middle lobe, and all basal segments of the right lower lobe. FDG avid small to mildly enlarged non-necrotic nodes are seen at right paratracheal, pretracheal, precarinal, subcarinal, right hilar, and right inferior pulmonary ligament, and lower paraesophageal regions may be noted.

An 11-year-old boy was referred to our Paediatric Rheumatology Clinic for evaluation of progressive refractory vasculitis with renal disease. The child had been symptomatic for past 3 years, and evaluation performed at the referring center showed severe anemia, neutrophilic leukocytosis, elevated inflammatory markers, and hypertension with nephrotic range proteinuria without active urinary sediments. He was also noted to have low ejection fraction (20–22%). Suspecting underlying vasculitis, a CT angiogram was done, which showed concentric wall thickening of the abdominal aorta, celiac artery, right renal artery stenosis with compensatory hypertrophy of the left kidney, and severe stenosis of the right pulmonary artery. Considering a diagnosis of Takayasu arteritis, he was commenced on pulsed methylprednisolone followed by cyclophosphamide pulses with antihypertensive medications. As intermittent fever persisted with proteinuria, he was referred to our institute. CT angiography and positron emission tomography study are described in Fig. 3. He was worked up for viral etiologies such as CMV, EBV, HSV, which were negative. The screen of all his blood records revealed persistent thrombocytopenia. On detailed history, he had two episodes of acute febrile illness, which were treated as dengue fever due to thrombocytopenia in past. However, he never had major clinical bleeds or serious infections. Whole-exome sequencing revealed hemizygous pathogenic mutation in exon 2 of the WAS gene, c.256C>T, p. Arg86Cys. WASP expression was also reduced. He was started on mycophenolate mofetil and maintenance IVIg, and cotrimoxazole prophylaxis and was being planned for HSCT, however succumbed to his illness at home following sudden-onset respiratory distress.

### Patient 4 (2023)

Another 11-year-old boy was referred to our center with diagnosis of Takayasu arteritis when he presented with complaints of recurrent abdominal pain, and neck and left upper limb pain with features of claudication. CT angiography had shown thickening of bilateral common carotid artery, complete opacification of left subclavian, origin at narrowing of the celiac trunk, and complete opacification of superior mesenteric artery with collaterals from inferior mesenteric artery. Methotrexate and oral corticosteroids were initiated. He had been previously well, and did not have bleeding manifestations, infections, or eczema in past. On evaluation at our institute, thrombocytopenia and leukopenia were noted, and attribute to methotrexate toxicity. Withdrawal of methotrexate and folinic acid supplementation resulted in recovery of leukopenia; however, thrombocytopenia persisted raising the suspicion of WAS. Moreover, mean platelet volume (MPV) was low, and serum immunoglobulins showed elevated IgA and IgE with low IgM levels. Estimation of WASP showed normal expression of protein but genetic sequencing revealed variant in exon 1 c.103C>T p. Leu35Phe. Urgent HSCT was advised, but on screening of the family, both brothers were also found to be affected. He was initiated oral steroids and mycophenolate and is awaiting a haploidentical HSCT.

## Discussion

Besides the classical triad of eczema, immunodeficiency, and microthrombocytopenia, patients with WAS are known to exhibit a spectrum of immunological abnormalities. Our case series adds to the limited literature by presenting four pediatric patients with WAS who developed large vessel vasculopathy, involving the aorta and its major branches. Vasculopathies in WAS can manifest as thoracic aortic aneurysms and dissections (TAADs), coronary artery disease, ischemic strokes, and even death, as seen in our patients.

Sullivan et al. reported vasculitis in 13% of patients in their multi-institutional study on WAS (5). However, multicenter cohort study from India reported vasculitis in a smaller subset (9.5%), predominantly affecting small vessels although autoimmune manifestations were observed in 40% of the cohort (13). The earliest description of large vessel vasculopathy in WAS was given by Lau et al. who reported a 5-year-old boy with Takayasu arteritis affecting the abdominal and renal arteries (16). Later Pellier et al. described five children with WAS who developed aortic aneurysms, predominantly involving the thoracic aorta and abdominal aorta (17). Four of these cases were asymptomatic, with aneurysms detected incidentally following radiological imaging; only one presented with acute chest pain. A summary of previously published cases of large vessel vasculopathy in WAS is provided in Table 2. The low number of reported cases may reflect the impact of early diagnosis and timely HSCT. All four patients in our series were older than 10 years and had not undergone HSCT until then. Patients 1 and 2 were symptomatic; their diagnoses had been established earlier, but HSCT was not performed due to financial constraints. Patients 3 and 4 exhibited vascular symptoms without significant infection or bleeding tendencies and likely represent an milder phenotype. This is further supported by the presence of missense mutations in exons 1 and 2, which are categorized as class 1 variants.

Immune dysregulation is thought to underlie the predisposition to vasculitis in WAS. Immune complex deposition within vessel walls may lead to necrotizing vasculitis. Alternatively, infectious triggers are well documented in vasculitis, and WAS patients are vulnerable to a broad range of pathogens. Pellier et al. found varicella-zoster virus, EBV, and human herpesvirus 6 in aortic tissue from a patient with aortic aneurysm (17). Patients 1, 2, and 4 in our series had significant EBV viremia. Patients 1 and 3 also had a pulmonary infection, and considering India’s high tuberculosis prevalence, an extensive workup was done. Although *Mycobacterium tuberculosis* could not be isolated, empirical antitubercular therapy was initiated. Patient 1 also had generalized lymphadenopathy raising the suspicion of lymphoma, though biopsy could not be obtained. Vasculitis secondary to lymphoma was considered, and he was treated with high-dose IVIg and rituximab for EBV.

Vasculopathies have also been reported in other actinopathies. Mutations in the ACTA2 gene, encoding alpha-smooth muscle actin (α-SMA), are associated with TAADs and other vascular disorders (18, 19). The Arp2/3 complex, which is also implicated in actin regulation, when disrupted, can cause vasculitis and inflammatory complications. α-SMA is essential for vascular smooth muscle cell (SMC) contraction and vessel wall integrity. ACTA2 mutations lead to SMC dysfunction, resulting in vessel wall remodeling, oxidative stress, hyperplasia, and fibrosis (18, 19, 20). Likewise, members of the DOCK family have been known to modulate downstream effectors involved in rearrangements of the actin cytoskeleton (21). Vascular abnormalities involving great vessels have been reported in Hyper IgE syndromes secondary to DOCK8 deficiency (22, 23, 24, 25). Additionally, actinopathies can impair immune cell function contributing to immune dysregulation and thus inflammation seen in vasculitis.

Evidence guiding the management of aortic aneurysms in WAS is limited to case reports (Table 2). Ilowite et al. reported resolution of vascular involvement with methylprednisolone and cyclophosphamide in a patient with aortic aneurysm and valvular regurgitation (4). Johnston et al. used azathioprine and surgical aortic root replacement in a 17-year-old WAS patient, achieving favorable outcomes (8). Surgical repair has been successful in several cases (6, 9, 10, 11, 12, 13, 14, 15), but is associated with heightened bleeding and infection risk due to underlying thrombocytopenia and immunodeficiency. All four of our patients received immunosuppression in the form of pulse methylprednisolone, methotrexate (patient 2), cyclophosphamide (patient 3), mycophenolate (patient 4), and rituximab (patient 1) but was ineffective. Endovascular intervention/surgical repair was contemplated in patients 1 and 2; however, it could not be done due to severe thrombocytopenia and extensive disease. HSCT could not be pursued because of financial constraints and lack of matched donors.

## Materials and methods

This study was conducted in the Allergy Immunology Unit of the Advanced Pediatrics Centre, Postgraduate Institute of Medical Education and Research, Chandigarh, a tertiary care center in Northern India. We retrospectively reviewed the records of children diagnosed with WAS from January 2005 to June 2025 who presented with features of large vessel vasculopathy at the Pediatric Immunology clinic. Data on demographic details, clinical presentation, detailed family history, laboratory investigations, immunological profile, radiological findings, genetic results, and outcomes were collected and tabulated in an Excel sheet.

All patients underwent assessment of complete blood counts including estimation of MPV by automated analyzers (COULTER HmX AL Analyzer, Beckman Coulter; or COULTER LH780 Hematology Analyzer, Beckham Coulter) standardized for MPV estimation. The MPV value between 7.5 and 11 fl was considered normal, and microplatelets were defined if MPV was <7.5 fl.

### Immunological evaluation

Serum IgG, IgM, and IgA were estimated by nephelometer (MININeph, semiautomated nephelometer, The Binding Site), while serum IgE was estimated by enzyme immunoassay. Surface immunophenotyping of lymphocyte subsets was performed on peripheral blood using fluorochrome-conjugated monoclonal antibodies. T cells were identified using anti-CD3-phycoerythrin (PE)-Cy7 (Becton Dickinson, BD), B cells with anti-CD19-fluorescein isothiocyanate (FITC) (BD), and natural killer (NK) cells with anti-CD56-APC/CD16-APC (BD). Within the CD3^+^ T cell population, helper (CD4-FITC) and cytotoxic (CD8-APC) subsets were delineated. Regulatory T cells were defined phenotypically as CD4^+^CD25^+^CD127^−^ cells. Data acquisition was performed on a BD LSRFortessa flow cytometer (BD Biosciences), and analysis was conducted using FlowJo software (Tree Star Inc.).

### WAS protein estimation

Intracellular staining of WASP using PE-labeled anti-human WASP antibody (sc-13139, PE [clone: B-9], Santa Cruz Biotechnology) was carried out on nonerythroid blood cells derived from the peripheral blood. Cells were gated using side scatter vs. CD45 labeled with FITC (555482; BD, A07782; Beckman Coulter Life Sciences). Lymphocytes were acquired on a flow cytometer (BD LSRFortessa). Median fluorescence intensity and stain index in stimulated and unstimulated samples were calculated.

### Genetic evaluation

Genetic tests were carried out to confirm the diagnosis after obtaining informed consent from parents or caregivers. Genomic DNA was isolated from peripheral blood samples using Qiagen kits (QIAamp DNA Blood Mini Kit, 51106; Qiagen Ltd.). A targeted next-generation sequencing was performed for primary immnue deficincy disease (PID) patients using a gene panel comprising 44 genes including the WAS gene on the Ion S5 platform (Ion Torrent S5, Thermo Fisher Scientific). One patient (patient 4) underwent whole-exome sequencing through a commercial laboratory.

### Radiological evaluation

**Figure 4.**
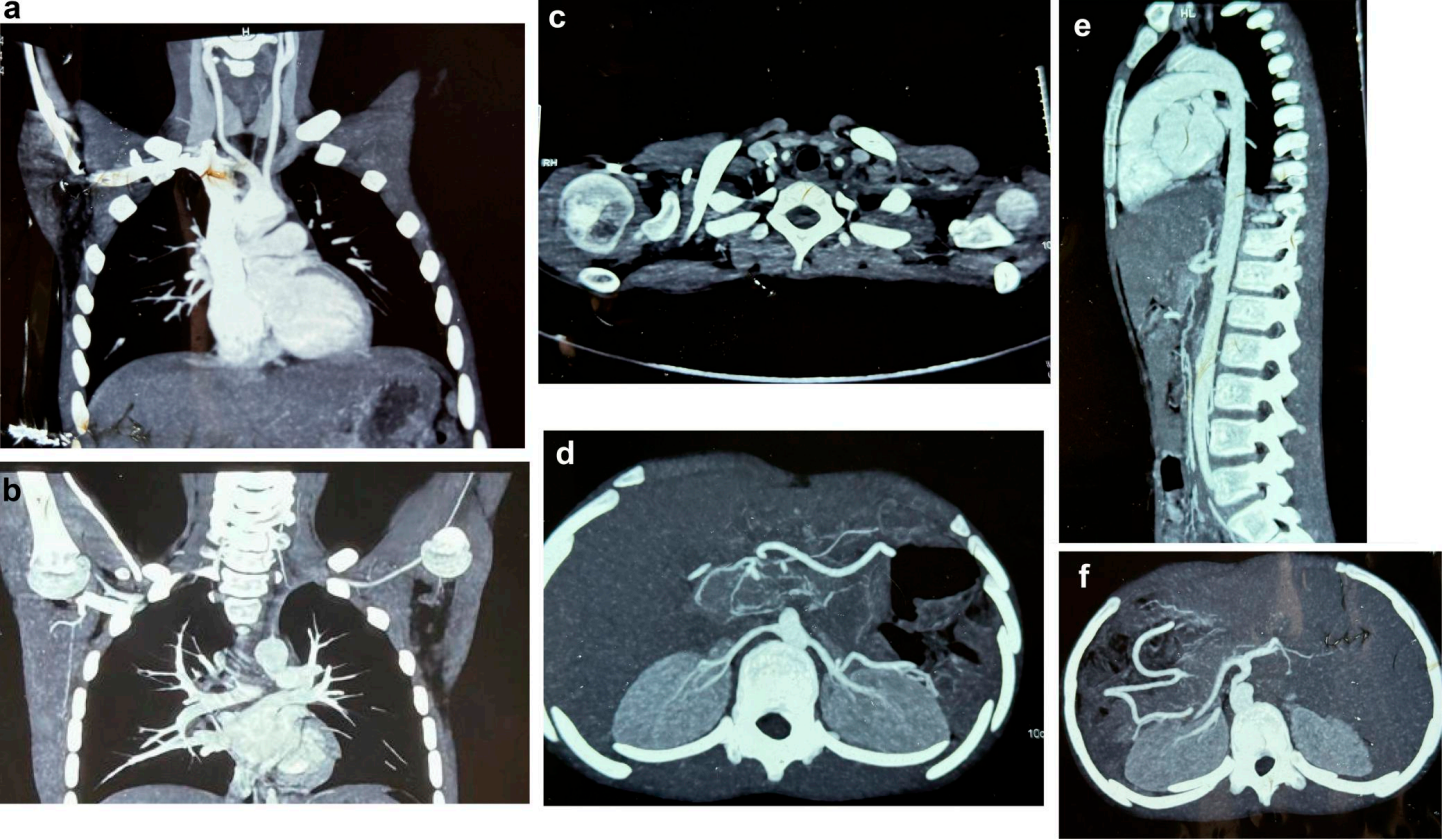
**Panel of CT angiograms showing extent of vascular involvement in patient 4. (a–f)** CT angiography of thoracic and abdominal vessels shows long segmental occlusion/tight stenosis of the entire length of the left subclavian artery with associated mild circumferential mural thickening. Left vertebral and left axillary arteries show attenuated caliber. Mild circumferential mural thickening involving the right and left common carotid arteries from the origin to bifurcation (c). Aortic root, ascending aorta, arch of aorta, descending thoracic aorta, abdominal aorta and its branches, bilateral common iliac arteries, bilateral external iliac arteries, and bilateral internal iliac arteries are normal in caliber and opacification (d–f).

Computed tomography angiography was performed in-house using a 192-detector dual-source CT scanner (Siemens SOMATOM Force), except for patient 1, whose scan was performed externally. Scans were acquired in the systemic arterial phase following intravenous administration of nonionic iodinated contrast. Image acquisition parameters were optimized for pediatric patients to minimize radiation exposure while maintaining diagnostic quality. Multiplanar and three-dimensional reconstructions were generated for detailed assessment of the aorta and its major branches. Figs. 1, 2, 3, and 4 are composite images illustrating features of vascular involvement. In some panels, scale bars could not be provided due to the process of creating composite figures.

To conclude, vasculitis in WAS tends to be progressive and destructive, and relapses have been noted. As many patients may remain asymptomatic, vasculitis may be underdiagnosed. Although most reported cases are in older patients, there are also reports of early-onset disease during infancy. Incidental discovery of WAS gene mutation in two of our patients with Takayasu arteritis with milder phenotype adds up to the spectrum of manifestations. This article highlights the importance of early screening and timely HSCT in all WAS patients, including those with mild or atypical features, to reduce the risk of vasculopathy.

## Ethics statement

This study was performed in line with the principles of the Declaration of Helsinki. The Departmental Review Board of the Advanced Pediatrics Centre, Postgraduate Institute of Medical Education and Research, Chandigarh, approved the manuscript (DRB87/25 dated 05/09/25), and informed consent was obtained from the parents of the children included in this study.

## Data Availability

Data are available on request to the corresponding author.
